# On Restoring Consciousness

**Published:** 1899-05

**Authors:** 


ARTICLE II.

### ON RESTORING CONSCIOUSNESS.

DR. BOWLES.

In all unconscious, comatose or syncopal conditions, however subtle or obscure the cause may be, whether from accident or from disease, whether from concussion or compression, whether from alcohol poisoning or from drugs, whether from suffocation or from drowning, or from any other cause, we have a condition of (in a physical sense) practical death of the body; a condition in which the patient cannot lift a finger to avert impending death, so that, if he is to be saved, it must be done with the help of those around him; he is now as much subject to the force of gravity as the dead bodies upon which we experimented in the post-mortem room, and if there are physical difficulties in the way interfering with respiration or circulation, they must at once be removed. Anesthesia is a condition essentially belonging to this category, but it is clinically often complicated by a mixed condition of syncope and apnea, and it is by no means easy to separate the one from the other.

It is clear that in the syncope of anesthesia the smallest interference with respiration is an instant danger; for to relieve lungs already surcharged with chloroform vapor we desire, in the first place, the expulsion of the noxious contents and to replace them with pure air. One's natural instinct is to fly to artificial respiration at once; but let us beware at such a moment of what a friend of mine calls "the vigor of fright."

In syncope the greatest care must be taken to avoid disturbance or pulling about of the body; if the ghost of a breath is being taken, wait and watch; a person nigh unto death wants very little air, metabolism is almost at a standstill, and as much air as the patient wants at that moment nature will supply. If we wish to prevent the extinction of dying embers, we do not violently disturb them by "poking the fire," or we dissipate heat, and so extinguish the spark that kept them alive. I have seen cases of illness in which it was certain that physical disturbance during syncops put the patient in imminent peril by calling on the heart for extra effort at a moment when that effort was impossible.—*British Medical Journal.*

